# Comparison of the Molecular Responses of Tolerant, Susceptible and Highly Susceptible Grapevine Cultivars During Interaction With the Pathogenic Fungus *Eutypa lata*

**DOI:** 10.3389/fpls.2019.00991

**Published:** 2019-07-30

**Authors:** Chloé Cardot, Gaetan Mappa, Sylvain La Camera, Cécile Gaillard, Cécile Vriet, Pascal Lecomte, Gérald Ferrari, Pierre Coutos-Thévenot

**Affiliations:** ^1^SEVE, Laboratoire Sucres & Echanges Végétaux-Environnement, UMR EBI, CNRS 7267, Université de Poitiers, Poitiers, France; ^2^INRA, UMR 1065 SAVE (Santé et Agroécologie du Vignoble), Université de Bordeaux, Villenave d’Ornon, France; ^3^BNIC (Bureau National Interprofessionnel du Cognac – Station Viticole), Cognac, France

**Keywords:** grape, eutypa dieback, *Eutypa lata*, cultivar susceptibility, pathogenesis-related (PR) proteins, stilbenes, sugar transporters, cell wall invertase

## Abstract

*Eutypa lata* is the causal agent of eutypa dieback, one of the most destructive grapevine trunk disease that causes severe economic losses in vineyards worldwide. This fungus causes brown sectorial necrosis in wood which affect the vegetative growth. Despite intense research efforts made in the past years, no cure currently exists for this disease. Host responses to eutypa dieback are difficult to address because *E. lata* is a wood pathogen that causes foliar symptoms several years after infection. With the aim to classify the level of susceptibility of grapevine cultivars to the foliar symptoms caused by *E. lata*, artificial inoculations of Merlot, Cabernet Sauvignon, and Ugni Blanc were conducted over 3 years. Merlot was the most tolerant cultivar, whereas Ugni Blanc and Cabernet Sauvignon exhibited higher and differential levels of susceptibility. We took advantage of their contrasting phenotypes to explore their defense responses, including the activation of pathogenesis-related (*PR*) genes, oxylipin and phenylpropanoid pathways and the accumulation of stilbenes. These analyses were carried out using the millicell system that enables the molecular dialogue between *E. lata* mycelium and grapevine leaves to take place without physical contact. Merlot responded to *E. lata* by inducing the expression of a large number of defense-related genes. On the contrary, Ugni Blanc failed to activate such defense responses despite being able to perceive the fungus. To gain insight into the role of carbon partitioning in *E. lata* infected grapevine, we monitored the expression of plant genes involved in sugar transport and cleavage, and measured invertase activities. Our results evidence a coordinated up-regulation of *VvHT5* and *VvcwINV* genes, and a stimulation of the cell wall invertase activity in leaves of Merlot elicited by *E. lata*, but not in Ugni Blanc. Altogether, this study indicates that the degree of cultivar susceptibility is associated with the activation of host defense responses, including extracellular sucrolytic machinery and hexose uptake during the grapevine/*E. lata* interaction. Given the role of these activities in governing carbon allocation through the plant, we postulate that the availability of sugar resources for either the host or the fungus is crucial for the outcome of the interaction.

## Introduction

Grapevine is a crop of great economic importance worldwide. Varieties used in all European vineyards belong to the *Vitis vinifera* species. In France, several grapevine varieties are imposed by French legislation, especially for the French AOC label (controlled designations of origin). For example, the main grapevine variety cultivated in the vineyard of Cognac (Charentes – France) is Ugni Blanc because of its high productivity trait and its lower susceptibility to the gray mold disease (personal communication, BNIC) (Cardot, 2017). This cultivar produces wines with low alcohol content and high total acidity, which is therefore ideal for the distillation of cognac spirits, but is highly susceptible to diseases, such as grapevine trunk diseases (GTDs; Péros, 1995; Dubos, 2002; Bertsch et al., 2013; Bruez et al., 2013). Collectively, the three main GTDs, esca disease, botryosphaeria dieback, and eutypa dieback, are major issues causing severe economic losses in vineyards (Sosnowski and McCarthy, 2017) by affecting plant vigor, reducing yield and quality of grapes, and premature plant death (Gramaje et al., 2018). For example, the cost of dead plant replacements due to these diseases is estimated to approximately 1.5 billion dollars per year worldwide (Hofstetter et al., 2012). GTDs have been associated with one or several fungal trunk pathogens, such as *Phaeomoniella chlamydospora*, *Phaeoacremonium minimum*, *Eutypa lata* or members of the Botryosphaeriaceae, which colonize vivaceaous parts of the plant at all stages of growth (Fontaine et al., 2016). Symptoms, which include both foliage and vascular tissues, can overlap among different GTDs, making difficult the identification in the field (Gubler et al., 2005; Surico et al., 2006; Gramaje et al., 2018). Apart from prophylaxis methods, there is currently no effective treatment to combat wood diseases (Mondello et al., 2017). The severity of symptoms vary among cultivars (Gubler et al., 2005; Marchi, 2006; Travadon et al., 2013), however, no resistance locus against GTDs have yet been identified in *V. vinifera*. Together with the progressive restriction in pesticides use by the European Union, research efforts are needed to find effective solutions to this issue (Cardot, 2017).

*Eutypa lata* is the main fungus responsible for the eutypa dieback disease but it can be recovered from wood lesions in combination with other fungi that may be associated with processes leading to esca disease (Péros et al., 1999). This ascomycete develops in the trunk and spreads with the release of ascospores during rainy and windy periods and by infecting pruning wounds (Moller and Carter, 1965; Sosnowski et al., 2007; Rolshausen et al., 2008). Eutypa dieback symptoms, which mostly occurs several years after the infection, include sectorial necrosis in trunk, stunted and weak shoots with small chlorotic leaves. Sectorial necrosis cause the rupture of sap flow resulting in the dieback of infected grapevines, and conduct to the death of the vine within 3–5 years (Larignon, 2012). Because the fungus is restricted to the wood cane and not detected in the herbaceous part, symptoms observed on leaves and young stems are notably due to the secretion and the translocation of fungal toxins such as eutypine, glycoproteins or cell wall degrading enzymes (Deswarte et al., 1996; Mahoney et al., 2003; Octave et al., 2006, 2008; Rolshausen et al., 2008; Andolfi et al., 2011; Morales-Cruz et al., 2015).

Overall, *V. vinifera* cultivars are greatly affected by a large number of pathogens (fungi, bacteria, oomycetes or viruses). Grape, like other plants, attempt to respond by activating defense mechanisms, such as antioxidant system, phenylpropanoid pathway, pathogenesis-related (PR)-proteins and phytoalexin production (Armijo et al., 2016). However, this arsenal of defense deployed by the host against aggressors is frequently not sufficient to counteract the disease development. Depending on the pathogen lifestyle (necrotroph, biotroph or hemibiotroph), different hormone-mediated signaling pathways regulate a transcriptional reprogramming and more largely plant defense mechanisms (Oliver and Ipcho, 2004; Glazebrook, 2005; van Kan, 2006). Based on *Arabidopsis thaliana* studies, it is generally admitted that jasmonic acid (JA) and ethylene (ET) mediate defense responses to necrotrophic pathogen, while salicylic acid (SA) is required for the resistance against biotrophs and hemibiotrophs, with antagonistic relationships between both pathways (Glazebrook, 2005). The phytohormone network consisting of JA, ET, and SA, is required for the two modes of plant immunity, pattern-triggered immunity (PTI), and effector-triggered immunity (ETI) (Jones and Dangl, 2006; Cui et al., 2015; Peng et al., 2018).

Several PR proteins, belonging to different classes described by van Loon et al. (2006), are synthesized in grapevine following infection through the recognition of MAMPs (Microbe-Associated Molecular Patterns) or DAMPs (Damage-Associated Molecular Patterns) like oligosaccharide, lipid and proteinaceous elicitors (Kortekamp, 2006; Gomès and Coutos-Thévenot, 2009). Most of the PR-proteins have direct antimicrobial properties (e.g., osmotin and thaumatin) through hydrolytic activities on pathogen cell wall (e.g., glucanase and chitinase), and/or indirectly leads to the production of elicitors which trigger additional defense responses (van Loon et al., 2006). Numerous studies have described the selective expression of PR-protein-encoding genes in various grapevine cultivars following infection with a wide range of pathogens, such as *Botrytis cinerea*, *Plasmopara viticola*, *Erysiphe necator* or *Pseudomonas syringae* pv *pisi* (Kortekamp, 2006; Chong et al., 2008; Armijo et al., 2016). Following infection with wood decay disease fungi, grapevine defense responses like PR-proteins synthesis and phytoalexins production are induced both at local (wood) and systemic (leaf, berry) levels [reviewed in Fontaine et al. (2016) and Bertsch et al. (2013)]. For example, Camps et al. (2010) reported an up-regulation of several genes encoding PR-proteins (thaumatin and osmotin, chitinase and β-1,3-glucanase) in leaves of infected rooted cuttings (Carbernet-Sauvignon) artificially infected with *E. lata*. This result has been further confirmed by the work of Mutawila et al. (2017) that showed that the elicitation of cell suspension culture of *V*. *vinifera* cv Dauphine with *E. lata* culture filtrate resulted in an induction of *VvPR2* (β-1,3-glucanase), *VvPR5* (thaumatin and osmotin-like proteins), *VvPR3* and *VvPR4* (chitinase), and *VvPR6* (protease inhibitor, PIN).

Induction of secondary metabolites are commonly associated with defense and pathogenic responses (La Camera et al., 2004; Piasecka et al., 2015). Secondary metabolism is strongly induced after infection of grapevine by various pathogens, including *E. lata* (Langcake, 1981; Adrian et al., 1997; Amalfitano et al., 2000; Jeandet et al., 2002; Armijo et al., 2016). Several reports have indicated an upregulation of phenylalanine ammonia-lyase (PAL), which encodes the first enzyme of the phenylpropanoid pathway, as well as genes coding for enzymes of the flavonoid and stilbenoid pathways, chalcone synthase (CHS) and stilbene synthase (STS), respectively (Rotter et al., 2009; Mutawila et al., 2017). Stilbenes, such as ε-viniferin and resveratrol, are major phytoalexins in grapes. Resveratrol limits *E. lata* mycelium growth *in vitro* (Coutos Thévenot et al., 2001), but it is uncertain whether the accumulation of resveratrol and phenolic compounds limits *in vivo* wood colonization (Mutawila et al., 2017).

According to current knowledge concerning grapevine/*E. lata* interaction, grapevine exhibits some of the typical responses of the PTI, such as PR-protein synthesis and secondary metabolites accumulation, suggesting that this fungus is perceived by the host (Rotter et al., 2009; Fontaine et al., 2016). However, most of *V. vinifera* cultivars are susceptible to *E. lata*, which indicates that defense responses are not sufficient to limit infection (Mutawila et al., 2017).

Alongside these so-called classical defense responses, responses to pathogens include important changes in host carbon metabolism, a reduction of photosynthesis and an accumulation of hexoses in the apoplast (Berger et al., 2007; Bolton, 2009). Furthermore, several recent studies indicate that regulation of carbon partitioning and competition for apoplastic sugars between the plants and the pathogen play a critical role in determining the outcome of the interaction [reviewed in Naseem et al. (2017), Bezrutczyk et al. (2017), and Oliva and Quibod (2017)]. Indeed, as heterotrophic for carbon, pathogens need photoassimilates produced by the host during the photosynthesis as energy sources for growth and life cycle completion. On the other side, defending plants require sugars for metabolic needs of individual sinks and to sustain the increased metabolism activity necessary to fuel plant defense. Depending on the mode of colonization, biotrophic, and necrotrophic pathogens use different strategies to retrieve sugars from host. As a consequence, heterotrophic pathogens may represent an additional sink competing with other sinks for carbohydrates, thereby modifying source-sink partitioning through the plant (Lemoine et al., 2013).

To gain access to carbohydrate at the plant-pathogen interface, both partners possess their own sugar transport machinery, including sugar transporters and cell wall invertases (Veillet et al., 2016, 2017). We and other previously reported the involvement of plant high-affinity sugar transporters in the recovery of hexoses from the apoplasm. Members of the Sugar Transport Protein (STP) family are induced in response to several fungal and bacterial pathogens, this include AtSTP13 in Arabidopsis and STP13 homologs in wheat (Lr67) and grapevine (VvHT5) (Hayes et al., 2010; Lemonnier et al., 2014; Moore et al., 2015; Yamada et al., 2016). In Arabidopsis, AtSTP13 is a component of the PTI and contributes to the fungal and bacterial resistance (Lemonnier et al., 2014; Yamada et al., 2016). Because sugars seem to be preferentially taken up in the form of hexoses, regulation of carbon fluxes across plasma membrane is also dependent on the sucrose cleaving activity of cell wall invertases (CWINs). Hence, coordinated activities of CWIN and STP proteins have been reported in several plant pathogen interactions (Ruan, 2014; Tauzin and Giardina, 2014; Veillet et al., 2016). For instance, Arabidopsis *AtSTP4*/*Atβfruct1* and grapevine *VvHT5*/*VvcwINV* are induced in response to biotrophic fungal infection and *AtSTP13*/*AtCWIN1*(*Atβfruct1*) are concomitantly induced by *B. cinerea* (Fotopoulos et al., 2003; Hayes et al., 2010; Veillet et al., 2016, 2017). Study of a loss-of-function mutation of *AtCWIN1* demonstrated that the corresponding protein was responsible for the *Botrytis*-induced apoplastic invertase activity in leaves (Veillet et al., 2016). To date, no information are available on the involvement of sugar transporters and invertases during infection with *E. lata*. Camps et al. (2010) showed that the most abundant genes in leaves that were regulated during the asymptomatic phase of *E. lata* infection were associated with energy metabolism, especially with the light phase of photosynthesis. Such alteration of the photosynthetic system probably contribute to the decline in plant growth and vigor and affect the carbon partitioning and the source/sink relationships.

In the present study, we compared the molecular responses of three *V. vinifera* cultivars after challenge with *E. lata*, the causal agent of eutypa dieback. We analyzed their levels of susceptibility 1 year after artificial inoculation of rooted cuttings with *E. lata* and investigated their specific molecular responses using the millicell *in vitro* infection system. We showed that the relative tolerance of Merlot is supported by the activation of a set of defense-related (PR, oxylipin, and phenylpropanoid) genes deployed by the host. Our study also underlines the probable involvement of the SA pathway and the coordinated role of a cell wall invertase and a hexose transporter in this process. This latter result suggests that active extracellular sucrolytic machinery and hexose uptake may be advantageous for the host by promoting the availability of sugar resources.

## Materials and Methods

### *E. lata* Culture

*Eutypa lata* strain BX1-10 was used to inoculate the plants materials. This strain was retrieved from the vineyard of Bordeaux in 1992 and characterized for its virulence and ability to induce important foliar symptoms on infected grapevine cuttings (Péros and Berger, 1994; Camps et al., 2010). Mycelium was cultured in Potato Dextrose Agar (PDA-Becton-Dickinton) medium at 22^∘^C in the dark. Infections were performed using a 15 days-old culture old *E. lata* (Cardot, 2017).

### Plant Materials, Growth Conditions, and Infection

Three grapevine cultivars, Merlot (clone 181), Cabernet Sauvignon (clone 169), and Ugni Blanc (clone 480) were used. Canes were collected in the dormant season and stored at 4^∘^C until use. For re-hydration and disinfection, canes were immersed overnight in water with 2% bleach (2.6% of active chlorine). Canes were cut with a pruning shear to provide cuttings with 2–3 internodes (20 cm long) from which the lower bud was immediately removed. Cuttings were then placed in pot containing sand and installed on heating layers (20^∘^C) to promote rooting for 6 weeks in a greenhouse. They were regularly water-sprayed to maintain a high humidity. After the root formation and the appearance of first three leaves (about 15 cm high), cuttings were transferred in potting soil (Peaty universal substrate n^∘^4, Klasmann–Deilmann GmbH, Germany) and cultivated in a greenhouse thermoregulated with heating and cooling systems to maintain temperature between 19 and 24^∘^C.

### Infection of Grapevine Cuttings With *E. lat*a

**FIGURE 1:**
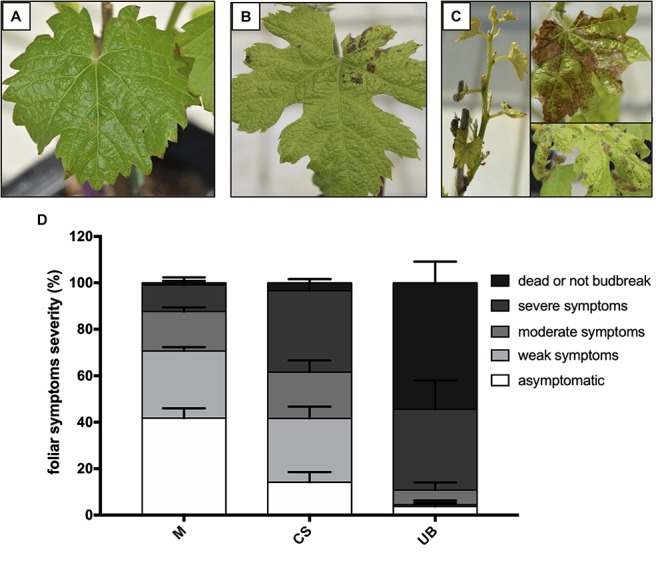
Comparison of foliar symptom severity between three grapevine cultivars. Eutypa dieback symptoms on Merlot (M), Cabernet-Sauvignon (CS), and Ugni Blanc (UB) were recorded on potted cuttings cultivated in greenhouse 1 year post-infection with the *E. lata* strain BX1-10. Five symptoms classes were established: asymptomatic plants (no symptoms and a normal growth), **(A)** weak symptoms (one spot on the whole plant), **(B)** moderate symptoms (some spots on 2–3 leaves), **(C)** severe symptoms (chlorosis, slow growth, several spots on the whole plant) and dead plant (dead plant or not bud break). The analysis was carried out on 120 infected plants (40 per year, during 3 years), and **(D)** percentage of each class was calculated. Pathogenicity tests were conducted over three consecutive years. Data from each year have been considered as biological replicates. Data presented are the mean of the percentage of each class of foliar symptom (*n* = 3). Error bars represent the standard errors of mean (SEM). Plants inoculated with sterile water have been used to control the absence of cross inoculation during the procedure. Results of the statistical analyses are shown in the Supplementary Table S1.

Sixty rooted cuttings (8 week old plants with 3–5 adult leaves) of each cultivar were used for the infection assay using a method previously developed by Chapuis (1995) and described by Camps et al. (2010) and Reis et al. (2019). Forty of these cuttings were infected with *E. lata* mycelium and twenty were inoculated with sterile water as control. Cuttings were drilled to create a 4 mm diameter hole at a 2 cm distance under the node. Infections were carried out by introducing a pellet of *E. lata* mycelium from a 15 days-old culture in PDA medium. The holes were then plugged with paraffin wax. Cuttings were maintained for 2 months in the greenhouse and then transferred in summer under a shelter-house until next spring. Foliar symptoms were assessed after bud break. A gradual scale of five classes has been determined using the following classification, which is similar to previous studies (Péros and Berger, 1994; Darrieutort and Lecomte, 2007; Camps et al., 2010; Cardot, 2017) (Figures 1A–C): asymptomatic plant (no typical symptom and normal growth), weak symptoms (sparse necrotic spots on one or few leaves), moderate symptoms (yellowing, spots on several leaves, reduced growth), severe symptoms (chlorosis, crispy leaves and short internodes, several spots on the whole plant) and dead plant or not bud break.

### *In vitro* Interaction Assays Between Foliar Discs From Grapevine Cuttings and *E. lata* Mycelium

Plants of each cultivars growing in greenhouse (University of Poitiers, France) were used to carry out molecular and biochemical analyses using the method described in Veillet et al. (2017) with some modifications.

*Eutypa lata* mycelium culture was prepared 4 days prior infection as follow: *E. lata* mycelium from 15 days-old culture in PDA medium was harvested from petri plates with 100 μL of sterilized water and mixed with 50 mL of Gamborg G0210 medium (3.16 g L^–1^, Duchefa biochemistry) containing sucrose (20 g L^–1^). The mycelium suspension was filtered (0.86 mm-20 mesh) to obtain a homogenous mixture. Three mL of the mycelium/Gamborg medium mixture were placed in 6-well *in vitro* culture plate (Dutscher) and incubated for 4 days at 22^∘^C under tridimensional shaking (50 rpm).

**FIGURE 2:**
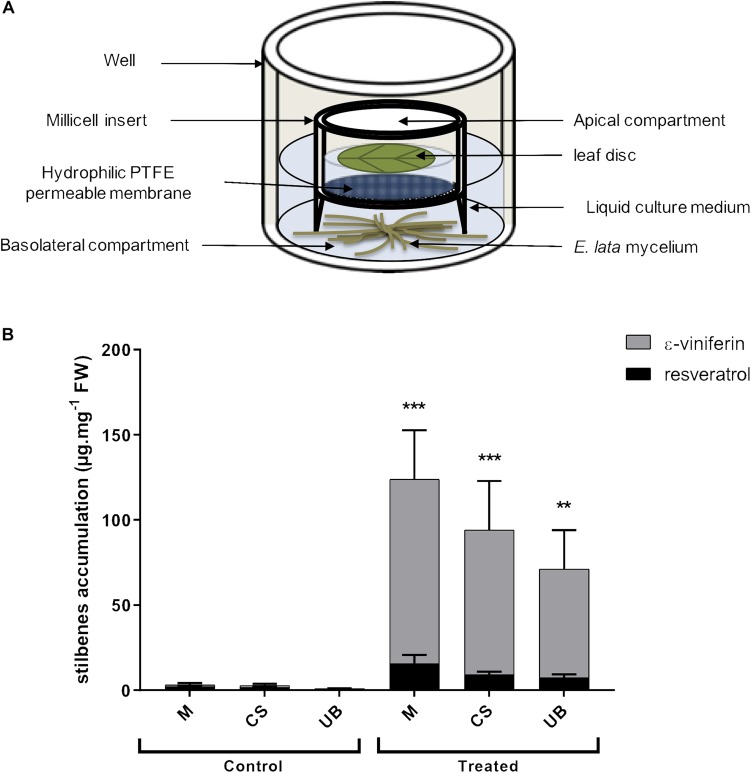
Establishment of the molecular dialogue between *E. lata* and grapevine leaf disc using the millicell infection system **(A)** Molecular communication between leaf disc and fungal mycelium (*E. lata*) is enable through the permeable membrane (0.4 μm pore diameter). Molecules secreted by leaf and fungus can pass through the membrane. This *in vitro* infection system is adapted from Veillet et al. (2017). **(B)** Stilbenes quantification by HPLC analysis in leaf disc and treated or not with *E. lata* using the millicell infection system. Data represent mean (±SEM) of eight independent experiments. Statistical analysis was performed using GraphPad Prism 7, with a Sidak’s multiple comparisons test between control and treated condition of ε-viniferin accumulation (^∗∗^*p* < 0.01, ^∗∗∗^*p* < 0.0001). No statistical difference has been determined between treated cultivars according to a Tukey’s multiple comparison test used to compare cultivars.

Leaves (3–5 foliar rank) from potted grapevine cultivar cuttings were collected, sterilized in a solution containing 5% NaOCl and 0.1% Tween 20 for 5 min and washed twice with sterilized water. Hydrophilic PTFE (Polytetrafluoroethylene) cell culture inserts (0.4 μm diameter pores, 30 mm diameter, Merck Millipore Ltd.) were placed in wells of 6-well culture plates containing growing mycelium. Three mL of fresh Gamborg/sucrose liquid medium were then added in each well. At time 0, a foliar disc of twenty-millimeter diameter was placed in the apical side of the millicell (treated condition) (Figure 2A). In the mock treatment (control condition), mycelium was omitted. Culture plates were incubated at 22^∘^C under a 16 h (light)/8 h (dark) photoperiod with tridimensional shaking (50 rpm) during 3 days and leaf discs were collected, dried and frozen in liquid nitrogen before being grinded using a tissue LyserII (Qiagen).

For benzothiadiazole (BTH) treatments, foliar discs were prepared as described above and incubated for 3 days in presence of BTH at a final concentration of 0.59 mM in 3 mL of Gamborg culture medium into a 6-well culture plate.

### Plant RNA Extraction and Reverse Transcription

Total RNA was extracted from frozen ground foliar discs. Leaf powder (100 mg) were mixed with extraction buffer (10 mL g^–1^ FW) containing 2% CTAB, 2% PVPP, 100 mM Tris–HCl pH 8, 25 mM EDTA, 2 M NaCl, 3.44 mM spermidine and 2% β-mercaptoethanol for 10 min at 65^∘^C. An equal volume of chloroform/isoamyl alcohol (24/1 v/v) was added and the mixture was centrifuged at 14,000 rpm for 10 min at 4^∘^C. This step was repeated once and the aqueous phase was collected. RNA was precipitated with 0.25 volume of 10 M LiCl overnight at 4^∘^C. RNA was pelleted by centrifugation at 15,000 rpm for 30 min at 4^∘^C and resuspended with a water solution containing 0.5% SDS. Samples were extracted with one volume of phenol/chloroform/isoamyl alcohol (25/24/1 v/v/v) and centrifuged at 14,000 rpm for 10 min at 4^∘^C. After an additional chloroform/isoamyl alcohol (24/1 v/v) extraction, 0.1 volume of sodium acetate 3 M and 2.5 of volume of ethanol (96%) were added to the supernatant and RNA were precipitated for 2 h at −20^∘^C. After centrifugation at 15,000 rpm for 30 min at 4^∘^C, RNA were washed with ice cold 70% ethanol, air dried and resuspended in 30 μL of sterilized water (Chang et al., 1993). RNA samples were quantified by spectrophotometry at 260 nm and their quality and integrity were verified by measuring the 260/280 nm ratio and by migration on agarose gel before DNase treatment (Sigma). For each sample, 2 μg of total RNA were reverse-transcribed in a total volume of 450 μL using M-MLV reverse transcriptase (Promega) following the manufacturer’s instruction. The cDNA obtained were stored at −20^∘^C (Cardot, 2017).

### Real-Time Quantitative Reverse Transcription-PCR (qRT-PCR) Analysis

Expression levels of 16 genes were analyzed by qRT-PCR. This gene set included 12 defense-related genes and four genes involved in sugar transport or metabolism (Supplementary Material S2). Primers were designed using the Primer3 tool (Untergasser et al., 2012) and tested for their specificity and efficiency (>95% for all primers). Real-time quantitative PCR was carried out using the GoTaq qPCR MasterMix (Promega) and a Mastercycler realplex2 instrument (Eppendorf). In this work, four *V. vinifera* reference genes were tested, elongation factor EF1γ and EF1*α*, glyceraldehyde-3-phosphate dehydrogenase (GAPDH) and actin, described in several previous reports (Reid et al., 2006; Nicolas et al., 2013; Camps et al., 2014). The stability of all these genes was tested in our biological conditions. GAPDH was finally selected as the best reference gene. Gene expression results were normalized to GAPDH as an internal standard (Supplementary Material S2). Results were expressed as relative gene expression using the 2^–Δ^*^*Ct*^* method (Schmittgen and Livak, 2008; Cardot, 2017). For all qRT-PCR analyses, the number of independent biological replicates is specified in the legend caption. For each independent experiment, three technical replicates have been performed.

### Extraction and Quantification of Phenolic Compounds by HPLC

Stilbenes were extracted with 100% methanol (250 μl per 100 mg FW of foliar discs), and methanolic extract was centrifuged at 20,000 × *g* for 5 min. This extraction was repeated twice. The supernatant was passed through a Sep-Pack^®^ C18 cartridge (Waters) to remove chlorophylls. Resveratrol and ε-viniferin were quantified by HPLC (Perkin Elmer 200 serie) according to a modified protocol of Coutos Thévenot et al. (2001). 180 μl of sample were loaded onto a silica C18 reverse phase column (Thermo Quest Hypersil Division, 5 μM, 250 × 4.6 mm) equilibrated with solvent A containing acetonitrile/TFA/H_2_O (1/0.1/99 v/v/v) at a flow rate of 0.8 mL min^–1^. Molecules are eluted with a step gradient of solvent B containing acetonitrile/TFA/H_2_O (90/10/0.1 v/v/v). The gradient is formed according to the following steps: 0–10 min, 35% of solvent B; 10–20 min, 50% of solvent B; 20–30 min, 80% of solvent B. Elution is monitored at 305 nm that corresponds to the optimal absorption wavelength of stilbenic compounds identified from retention time analysis with commercial standards. Resveratrol and ε-viniferin standards (Sigma) were used to establish the calibration curve as a function of integrated peak area. Results are expressed in μg of resveratrol or ε-viniferin g^–1^ fresh weight (Cardot, 2017).

### Extraction and Quantification of Invertase Activities

Invertases were extracted from grapevine leaves and enzymes activities were measured using the method described in Veillet et al. (2016) with some modifications. A total of 220 mg of powder leaves were mixed with 1400 μl of ice-cold extraction buffer (50 mM HEPES, 1 mM EDTA, 5 mM DTT, and 1 mM PMSF). Soluble and insoluble fractions were separated by centrifugation at 20,000 × *g* for 15 min at 4^∘^C. The supernatant contains cytoplasmic and vacuolar invertases, and the pellet contains cell wall invertases. The pellet was washed four times and resuspended in 700 μl of extraction buffer. To measure invertase activity, 50 μL of cell wall pellet suspension or soluble extract were mixed with 400 μl acetate buffer (100 mM) containing sucrose (100 mM v/v (pH 4 for apoplastic activity or pH 5 for vacuolar activity) or HEPES buffer (25 mM) containing sucrose (100 mM) (pH7 for cytoplasmic activity), and incubated 60 min at 30^∘^C. To stop the reaction, 500 μl of DNSA reagent (1% 3.5-dinitrosalicylic acid, 0.5 M KOH and 1 M sodium potassium tartrate) were added and the mixture was placed in a boiling-water bath for 10 min and then cooled to room temperature. Tissue debris were removed by centrifugation at 20,000 × *g* for 5 min. Absorbance was read at 560 nm using a microplate reader (Multiskan GO, Thermo Fisher). Results were normalized to the fresh weight (Cardot, 2017).

### Statistical and Computer-Assisted Analysis

Statistical and computer-assisted analyses were performed using the GraphPad Prism version 7.00 (GraphPad Software^1^, La Jolla, CA, United States). Pathogenicity tests were conducted over three consecutive years. Results from each year has been considered as biological replicate. Data presented are the mean of the percentage of each class of foliar symptom (*n* = 3). Plants inoculated with sterile water have been used to control the absence of cross inoculation during the procedure. Because they were not inoculated with *E. lata*, they were not integrated in the statistical comparison of the symptom severity between cultivars. For each class of symptoms, percentages of foliar symptom severity of Merlot, Cabernet Sauvignon, and Ugni Blanc cultivars have been compared using a Sidak’s multiple comparisons test.

For expression analyses, stilbene quantification and enzyme activities, comparisons between control and treated conditions have been analyzed using a Sidak’s multiple comparisons test. For control or treated conditions, statistical differences between cultivars have been analyzed using a Tukey’s multiple comparison test.

## Results

### The Grapevine Cultivars Merlot, Cabernet Sauvignon, and Ugni Blanc Exhibit Different Levels of Susceptibility to the Fungal Pathogen *E. lat*a

With the aim to classify the level of susceptibility of Merlot (M), Cabernet Sauvignon (CS), and Ugni Blanc (UB) cultivars to *E. lata*, we performed artificial inoculations of plants from rooted cuttings and followed eutypa dieback symptoms after 1 year. As seen in Figure 1 and Supplementary Table S1, Merlot, Cabernet Sauvignon, and Ugni Blanc cultivars displayed contrasting disease susceptibility phenotypes, while none of the 60 control plants of each cultivar, that were not inoculated with *E. lata*, showed symptoms of eutypa dieback. In our conditions, the Merlot cultivar was the most tolerant to eutypa dieback, as 38% of inoculated plants were asymptomatic and a majority of symptomatic plants exhibited weak (30%) or moderate symptoms (17%) after 1 year (Figure 1B). Among the 120 inoculated plants, only 18 plants were severely affected by the disease and only one plant was dead. In contrast, Ugni Blanc cultivar exhibited a very high level of susceptibility to *E. lata*, as 87% of inoculated plants were dead or displayed severe symptoms (53 and 24%, respectively) after 1 year. Only one plant showed weak symptoms and five plants were asymptomatic. In comparison with Merlot and Ugni Blanc, Cabernet Sauvignon cultivar exhibited an intermediate phenotype (Supplementary Table S1). According to the disease scoring, this cultivar can be considered as susceptible, as most of the infected plants displayed symptoms with different degrees of severity and only few inoculated plants were asymptomatic (14%).

In order to correlate the presence of *E. lata* in the cane with foliar symptoms, *E. lata* was re-isolated *in vitro* from cane segments 1 year after the inoculation. Accordingly, the presence of *E. lata* was detected in all symptomatic plants of the three cultivars (data not shown), thus validating our infection procedure.

We were able to show that Merlot, Cabernet Sauvignon, and Ugni Blanc were differentially affected by *E. lata* in controlled conditions. We took advantage of the contrasting susceptibility of these three cultivars to explore their specific molecular responses.

### Establishing the Molecular Dialogue Between *E. lata* and Grapevine Leaf Cells Using the Millicell *in vitro* Infection System

Because *E. lata* is a wood pathogen that is not found in leaves, it implies that long distance signals, released by the fungus in the wood, reach the leaves. To study such mechanisms, we developed the millicell system (Veillet et al., 2017), which allows the molecular dialogue between living organisms without physical contact. Here, the millicell insert physically separates growing *E. lata* mycelium (basolateral compartment) and grapevine leaf disc (apical compartment), but enabled selective flow of molecules secreted by both partners across a membrane (Figure 2A).

To verify the molecular dialogue was established between *E. lata* and grapevine leaf discs after 3 days of interaction, we monitored the accumulation of ε-viniferin and resveratrol in all three grapevine cultivars described above. These stilbenes represent major forms of phytoalexins in grape and may be good markers of the fungus recognition by the host (Adrian et al., 1997; Coutos Thévenot et al., 2001; Jeandet et al., 2002, 2013; Mutawila et al., 2017). In untreated leaf discs (control), levels of resveratrol and ε-viniferin were below the detection threshold, indicating that leaf discs were not affected after 3 days in the millicell without *E. lata*. In contrast, the content of ε-viniferin, and to a lesser extent resveratrol, increased strongly upon elicitation by *E. lata* (treated) in all three cultivars (Figure 2B). Moreover, we observed that the content of stilbenes tends to decrease with the cultivar susceptibility even though the difference between treated cultivars were not statistically significant (Figures 1B, 2B).

These results suggest that elicited leaf cells perceive the fungus through the release of pathogen or damage-associated molecular patterns (PAMPs or DAMPs) and trigger PAMP/DAMPs-induced responses, such as the accumulation of phytoalexins. Moreover, no symptom was visible in leaf discs after 3 days of interaction with *E. lata* secretome indicating that the millicell is a suitable system to monitor early molecular responses in grapevine elicited leaves.

### PAMP/DAMPs-Triggered Responses of Grapevine Cultivars With Differential Susceptibility to *E. Lat*a

We took advantage of the millicell system, which imposes strictly controlled conditions of elicitation, to compare general defenses of Merlot, Cabernet Sauvignon, and Ugni Blanc cultivars, previously described as differentially affected by *E. lata* (Figure 1). We analyzed the relative expression levels of seven genes encoding PR proteins in leaves, chosen as representative of different defense signaling pathways in grapevine (Jacobs et al., 1999; Robert et al., 2001; Aziz et al., 2003, 2004; Liu and Ekramoddoullah, 2006; Chong et al., 2008; Giacomelli et al., 2012; Spagnolo et al., 2017; Stempien et al., 2017).

**FIGURE 3:**
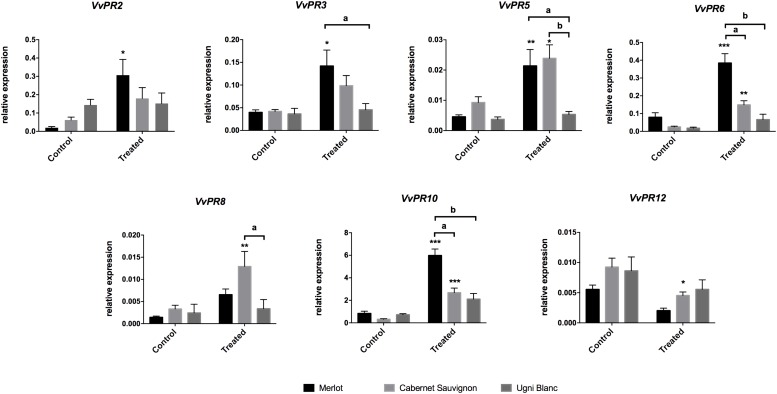
PR-protein gene expression in response to *E. lata* infection in Merlot, Cabernet Sauvignon, and Ugni Blanc cultivars. Relative expression of *VvPR2* (β 1–3 glucanase), *VvPR3* (chitinase class I), *VvPR5* (thaumatin-like), *VvPR6* (protease inhibitor), *VvPR8* (chitinase class III), *VvPR10* (ribonuclease-like), and *VvPR12* (defensin-like) genes. Relative gene expressions were analyzed for the Merlot, Cabernet Sauvignon, and Ugni Blanc leaf discs in control and treated (elicited) conditions using the Millicell system. Data represent mean (±SEM) of six independent experiments. Statistical analysis was performed using GraphPad Prism 7. Stars represents significant difference between control and treated condition for each cultivar, with a Sidak’s multiple comparisons test (^*^p < 0.05, ^∗∗^*p* < 0.001, ^∗∗∗^*p* < 0.0001). Letters represent results of a Tukey’s multiple comparison test used to compare cultivars.

In untreated leaves, basal transcript levels of all defense-related genes were low and relatively similar between cultivars (Figure 3). After 3 days of elicitation, comparison of expression patterns revealed that defense-related genes were differentially expressed among cultivars (Figure 3). Merlot challenged with *E. lata* exhibited a significant induction of 5 out of 7 *PR* genes, which belong to different classes, namely *VvPR2* (β-1,3-glucanase), *VvPR3* (chitinase class I), *VvPR5* (osmotin and thaumatin-like), *VvPR6* (protease inhibitor, PIN), and *VvPR10* (ribonuclease-like). These genes were induced 17, 3, 5, 7, and 10 times, respectively. Moreover, transcript levels were very high for *VvPR10* and relatively less abundant for *VvPR2* and *VvPR6*. In Cabernet Sauvignon, 4 *PR* genes were up-regulated by *E. lata*, i.e., *VvPR5*, *VvPR6*, *VvPR8* (chitinase class III) and *VvPR10*, whereas only *VvPR12* was down-regulated. Strikingly, the expression of all 7 *PR* genes was not significantly modified in the elicited Ugni Blanc compared to the control condition. The most tolerant cultivar, i.e., Merlot, responded to *E. lata* by inducing a large number of *PR* genes which belong to different classes, including proteins with antifungal activities (chitinase and glucanase) (van Loon et al., 2006). On the contrary, Ugni Blanc cultivar shown to be highly susceptible to *E. lata* was unable to activate such defense responses. Lastly, in agreement with its susceptibility phenotype, Cabernet Sauvignon exhibited intermediate responses.

**FIGURE 4:**
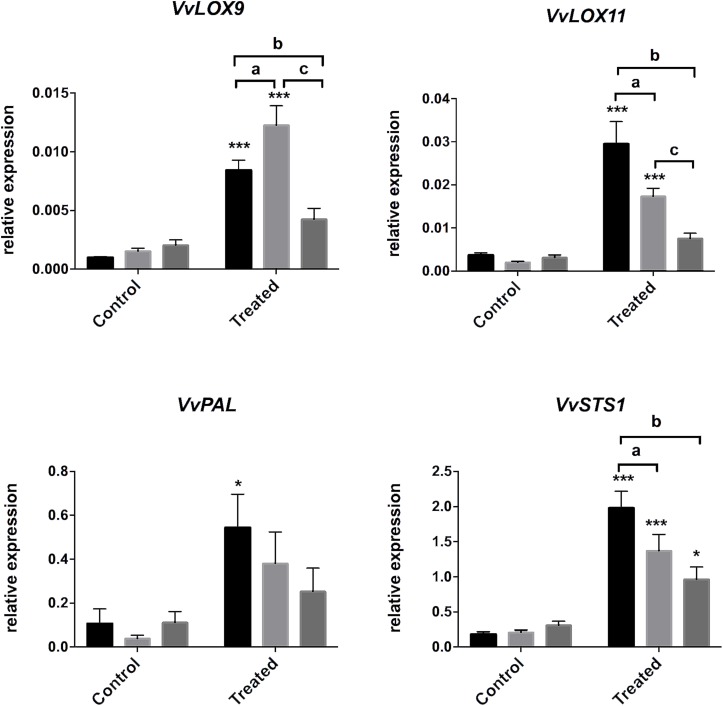
Gene expression in response to *E. lata* infection in Merlot, Cabernet Sauvignon, and Ugni Blanc cultivars. Relative expression of genes of lipoxygenases 9 and 11 (*VvLOX9* and *VvLOX11*), phenyl alanine ammonia lyase (*VvPAL*), and stilbene synthase (*VvSTS1*). Relative gene expressions were analyzed in Merlot, Cabernet Sauvignon, and Ugni Blanc leaf discs in control and treated (elicited) conditions using the Millicell system. Data represent mean (±SEM) of six independent experiments. Statistical analysis was performed using GraphPad Prism 7. Stars represents significant difference between control and treated condition for each cultivar, with a Sidak’s multiple comparisons test (^*^p < 0.05, ^∗∗∗^*p* < 0.0001. *N* = 6, excepted for *VvSTS1*: *N* = 10). Letters represent results of a Tukey’s multiple comparison test used to compare cultivars.

To go further, we analyzed genes of the oxylipin pathway, *VvLOX9* and *VvLOX11*, that encodes lipoxygenases (Podolyan et al., 2010), and genes of the phenylpropanoid pathway, *VvPAL*, encoding the phenylalanine ammonia lyase, and *VvSTS1* encoding the STS responsible for the biosynthesis of resveratrol and stilbenoid compounds. In Merlot, *VvLOX9*, *VvLOX11*, *VvPAL*, and *VvSTS1* were overexpressed in treated leaves suggesting that both oxylipin and phenylpropanoid pathways were activated in this cultivar. Cabernet Sauvignon responded to *E. lata* by a significant increase of *VvLOX9*, *VvLOX11*, and *VvSTS1* transcript levels, but not *VvPAL.* No statistically significant change in expression was detected for these genes, except for *VvSTS1*, in Ugni Blanc upon challenge, which is in line with the absence of *PR* induction in this cultivar. It should be noted that *VvSTS1* is significantly induced in all cultivars, which is consistent with a transcriptional regulation of the stilbene production in infected leaf discs (Figures 2B, 4).

Altogether, our results indicate that *E. lata* is detected by grapevine leaf cells in the millicell system without physical contact, which implies the release of PAMPs, DAMPs or effectors from the fungus. The perception by the host of such molecules leads to the activation of defense signaling pathways, especially in Merlot which exhibited a relative tolerance to eutypa dieback disease. By investigating the molecular responses of cultivars with differential levels of susceptibility to *E. lata*, we showed that the degree of tolerance is supported by the activation of clusters of defense-related genes. Our data reveal that UB was able to perceive the presence of the fungus that is exemplified by the induction of the STS pathway. However, this highly susceptible cultivar failed to trigger efficient defense responses, such as the activation of *PR* gene expression.

### Involvement of Sucrose Cleavage and Hexose Transport Processes During the Grapevine/*E. lata* Interaction

**FIGURE 5:**
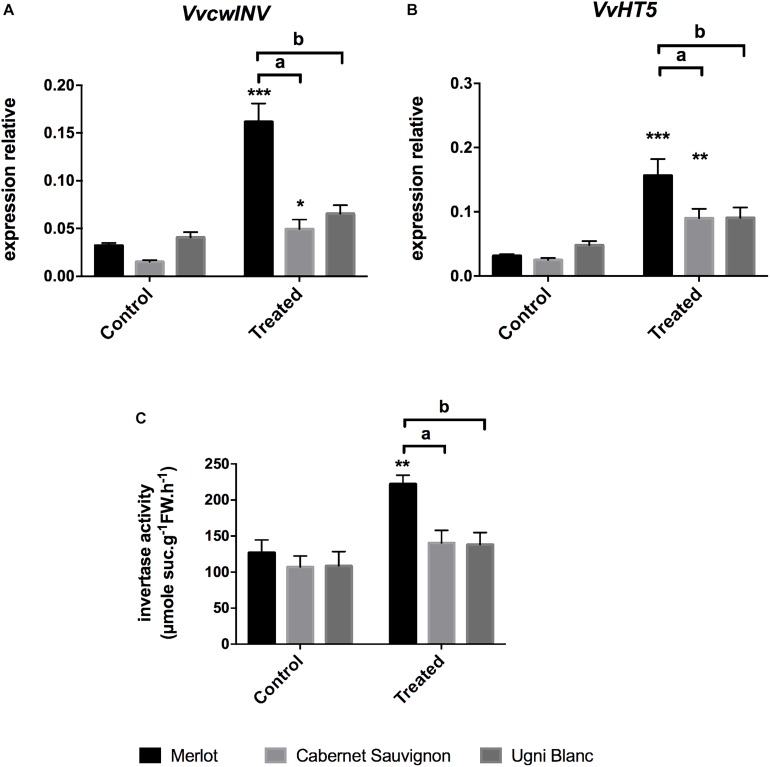
Sugar cleavage and transport-related gene expression, and cell wall invertase activity in response to *E. lata* infection in Merlot, Cabernet Sauvignon, and Ugni Blanc cultivars. **(A)** Cell wall Invertase (*VvcwINV*) and **(B)** hexose transporter (*VvHT5*, relative gene expression were analyzed by RT-qPCR in Merlot, Cabernet Sauvignon, and Ugni Blanc leaf discs in control and treated (elicited) conditions using the Millicell system. **(C)** Cell wall invertase activity was studied in control and treated foliar discs using the Millicell system. Data represent mean (±SEM) of six independent experiments for the invertase activity analysis and 10 independent experiments for the gene expression analysis. Statistical analysis was performed using GraphPad Prism 7.00. Stars represents significant difference between control and treated condition for each cultivar, with a Sidak’s multiple comparisons test (^∗∗^*p* < 0.001, ^∗∗∗^*p* < 0.0001). Letters represent results of a Tukey’s multiple comparison test used to compare cultivars.

Many studies suggest that pathogen infection leads to the establishment of a new sink competing with existing sinks (Lemoine et al., 2013). Cell wall invertases, which cleaves apoplastic sucrose into glucose and fructose, and plasma membrane hexose transporters are key regulators of sink strength by supporting sink demand for sugars and providing resources to cells responding to infections (Tauzin and Giardina, 2014). To gain insight into the putative role of the carbon partitioning in *E. lata* infected grapevine, we monitored the expression of *VvcwINV* and *VvHT5*, previously described as being involved in grapevine pathogenic responses, as well as sucrose degrading activities in leaves (Hayes et al., 2010). As seen in Figure 5, the cell wall invertase *VvcwINV* and the hexose transporter *VvHT5* exhibited similar expression patterns. These genes were significantly induced in Merlot (fivefold) and Cabernet Sauvignon (fourfold and threefold, respectively) leaf discs challenged with *E. lata*. In contrast, transcript levels of both genes were not significantly different in treated Ugni Blanc compared to corresponding controls.

In addition to the study of transcriptional responses, insoluble fractions of protein extracts were assayed for cell wall invertase activities to explore the impact of *E. lata* infection on carbon fluxes in all three grapevine cultivars. Elicitation of Merlot leaves led to a significant stimulation of the cell wall invertase activity that was increased by 176% after 3 days, whereas in susceptible cultivars (CS and UB), cell wall invertase activities were not changed (Figure 5C). We noticed that transcript accumulation of *VvcwINV* did not fully correlate with enzymatic activity. This result is in accordance with the probable involvement of post-translational regulation of cell wall invertases as suggested by the work of Veillet et al. (2016).

Because intracellular sucrolytic activities, i.e., cytosolic and vacuolar invertases, are important in regulating the intracellular sugar homeostasis (Roitsch and Gonzalez, 2004), we monitored the transcript accumulation of genes encoding cytosolic invertase (*VvCIN2*) and vacuolar invertase (*VvGIN2*) and the related cytoplasmic and vacuolar invertase activities in elicited leaves. Results indicates that intracellular invertases were functional in all cultivars but their sucrolytic activities were relatively less important than cell wall invertase (Supplementary Material S1 and Figure 5C). Ugni Blanc showed a very high vacuolar invertase activity, which may be due to its productive and vigorous metabolism. As shown in Supplementary Material S1, neither the level of transcript of *VvCIN2* and *VvGIN2*, nor the cytoplasmic and vacuolar invertase activities were modified upon elicitation.

Altogether, our results evidenced a coordinated transcriptional up-regulation of *VvcwINV* and *VvHT5* genes, accompanied by a stimulation of the cell wall invertase activity in leaves of merlot challenged by *E. lata.* It suggests that the enhanced extracellular sucrolytic machinery and hexose uptake may participate to the transition from source to sink upon infection.

### BTH-Responsive Expression of Genes in Tolerant and Highly Susceptible Grapevine Cultivars

**FIGURE 6:**
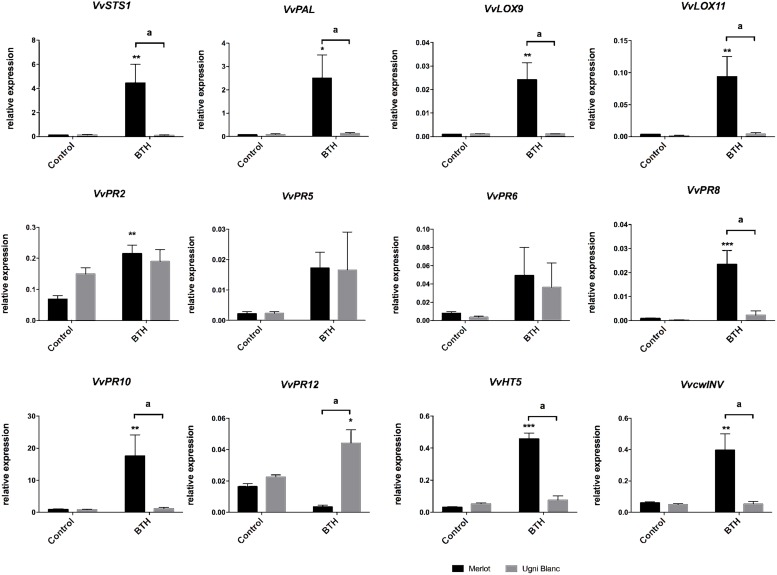
Sugar metabolism and defense gene expression in response to treatment with the SA analog BTH in Merlot and Ugni Blanc cultivars. Stilbene synthase (*VvSTS1)*, phenylalanine ammonia lyase (*VvPAL*), lipoxygenase 9 (*VvLOX9*), lipoxygenase 11 (*VvLOX11*), *VvPR2* (β 1–3 glucanase), *VvPR5* (thaumatin-like), *VvPR6* (protease inhibitor), *VvPR8* (chitinase class III), *VvPR10* (ribonuclease-like), *VvPR12* (defensin-like) genes, hexose transporter (*VvHT5*), and cell wall Invertase (*VvcwINV*) relative gene expressions were analyzed by RT-qPCR in Merlot and Ugni Blanc leaf discs in control and BTH-treated conditions using the Millicell system. Data represent mean (±SEM) of three independent experiments. Statistical analysis was performed using GraphPad Prism 7.00. Asterisk represent significant difference between control and elicited condition for each cultivar, with a Sidak’s multiple comparisons test (^*^p < 0.05, ^∗∗^*p* < 0.05, ^∗∗∗^*p* < 0.0001). Letters represent results of a Tukey’s multiple comparison test used to compare cultivars.

Many studies have reported that defense potentiators like β-aminobutyric acid (BABA), SA, methyljasmonate, laminarine sulfate or BTH, are effective against a broad spectrum of grapevine pathogens, such as *P. viticola* (downy mildew), *E. necator* (powdery mildew) or *B. cinerea* (gray mold) by strengthening plant defenses *via* the accumulation of PR proteins, phytoalexins, or phenolic compounds (Adrian et al., 1997; Jacobs et al., 1999; Aziz et al., 2003; Hamiduzzaman et al., 2005; Belhadj et al., 2008; Trouvelot et al., 2008; Dufour et al., 2013). BTH, an analog of SA, is a systemic acquired resistance (SAR) inducer that mimics the natural signaling activity of SA with no direct antifungal activity (Dufour et al., 2013; Bektas and Eulgem, 2014; Jiao et al., 2016). BTH treatment of Cabernet Sauvignon triggered the upregulation of several *PR* genes, *VvPAL*, *VvSTS1*, and *VvLOX9*, indicating that these genes were SA-inducible (Dufour et al., 2013). Because these defense-related genes were also induced by *E. lata*, we investigated whether the SA-inducibility of these genes were effective in leaf discs of Merlot and Ugni Blanc cultivars, described as tolerant and highly susceptible cultivars. In accordance, *VvSTS1*, *VvPAL*, *VvLOX9*, but also *VvPR2*, *VvPR8*, *VvPR10*, and *VvLOX11* were differentially expressed after BTH treatment in Merlot (Figure 6). Interestingly, BTH failed to induce all these genes in the leaves of Ugni Blanc. To go further, we monitored transcript levels of *VvcwINV* and *VvHT5* (Figure 6). Both genes followed the same expression patterns and were also differentially upregulated in Merlot (7 and 11 fold, respectively) but were not responsive to BTH in Ugni Blanc. *VvPR12*, encoding a defensin-like protein classically dependent on JA signaling (Penninckx et al., 1996; Manners et al., 1998; Giacomelli et al., 2012) displayed a significant induction by BTH treatment in UB but not in Merlot.

We showed that several defense- and sugar-related genes are induced by *E. lata* and are also responsive to SA analog in the tolerant Merlot, but not in Ugni Blanc. This result indicates that some components of the SA signaling, that are effective in Merlot, are deficient in the highly susceptible cultivar Ugni Blanc.

## Discussion

With the aim to explore biological processes involved in the mechanism of tolerance/susceptibility of *V. vinifera* against the wood decay fungus *E. lata*, we chose to undertake an analysis of molecular responses of three grapevine cultivars. To that end, grapevine cultivars with clear contrasting susceptibility to eutypa dieback were required. Available data on the susceptibility of *V. vinifera* cultivars to *E. lata* dieback are based on field observations by examination of foliar and/or wood symptoms and on artificial inoculations. Accordingly, cultivars have been ranked from tolerant to highly susceptible (Dubos, 1987, 2002; Péros and Berger, 1994; Chapuis et al., 1998; Fussler et al., 2008; Bertsch et al., 2013; Bruez et al., 2013). Such susceptibility scales have to be analyzed with caution, because susceptibility was designated through the leaf symptom intensity rather than the proportion of tissues killed by the fungus. However, besides varietal susceptibility, the degree of susceptibility of some cultivars can vary across studies because many other factors influence the disease development (region, climate, soil, grapevine age, strain genetic variations, presence of a other trunk pathogens…) (Munkvold, 1995; Péros, 1995; Péros et al., 1997; Chapuis et al., 1998; Sosnowski et al., 2007; Grosman and Doublet, 2012; Travadon et al., 2013; Lecomte and Bailey, 2015). For this reason, we have initiated a phenotypic analysis using rooted cuttings infections of three grapevine cultivars, Merlot, Cabernet Sauvignon, and Ugni Blanc, cultivated in semi-controlled conditions. These cultivars were chosen because they are representative of French regions of Bordeaux and Charentes. We found that Merlot, Cabernet Sauvignon, and Ugni Blanc exhibited clear contrasting phenotypes, with Merlot being relatively tolerant compared to Ugni Blanc, which was highly susceptible. Overall, these results are in accordance with the degree of susceptibility reported in previous studies. However, Cabernet Sauvignon was susceptible with an intermediate response, whereas it was rather classified as highly susceptible in previous reports (Péros and Berger, 1994; Péros, 1995; Travadon et al., 2013). The contrasting degrees of susceptibility of these grapevine cultivars to eutypa dieback represents a good opportunity to investigate their specific molecular responses in order to better understand underlying mechanisms governing the tolerance or susceptibility to this wood pathogen.

Early molecular responses to *E. lata* are difficult to study because experimental models (*in vitro*, greenhouse, or vineyard) are confronted with several technical issues. For example, infection process and environment are not controlled in vineyard and symptoms appear approximately after 1 year in artificially infected rooted cuttings. *In vitro* plants do not differentiate much woody tissue. Moreover, RNA isolation in woody tissues is hazardous. Cell suspension culture is a reproducible model system but responses of a single cell type-growing culture may be different from those of heterogenous tissues, such as leaf or stem tissues. However, previous reports, including such experimental models, brought valuable information on the transcriptomic responses of *V. vinifera* cv Cabernet Sauvignon during symptom expression and on the defense-related responses of *V. vinifera* cv Dauphine cell suspension to *E. lata* culture filtrate (Camps et al., 2010; Mutawila et al., 2017). Lastly, in wood decay diseases, the fungus is confined in the wood and is never found in leaves or herbaceous shoots, where symptoms are typically observed. This implies that interactions between the fungus and leaf cells occur through the systemic action of fungal secreted molecules or effectors. Consequently, we adapted the infection system described by Veillet et al. (2017), which enables a molecular dialogue between fungus and host cells across a semi-permeable membrane without physical contact, to study early foliar responses to *E. lata* secretome. In a previous work, we successfully used the millicell system to unravel the molecular and biochemical responses during *A. thaliana* and *B. cinerea* interaction. The so-called millicell system provides highly controlled conditions and reproducible results enabling the comparison of the grapevine cultivars previously described as differentially affected by *E. lata.* In order to prove that leaf discs perceive signals from *E. lata*, we monitored the accumulation of stilbenic compounds, ε-viniferin and resveratrol, which is a classical PAMP-induced response in grapevine that plays key roles in plant resistance to a wide range of pathogens (Langcake, 1981; Adrian et al., 1997; Amalfitano et al., 2000; Coutos Thévenot et al., 2001; Belhadj et al., 2008; Chang et al., 2011). In numbers of reports investigating grapevine responses to various pathogens, stilbene contents are often correlated with disease resistance (Chong et al., 2009). Here, a strong induction of both ε-viniferin and resveratrol contents has been observed upon *E. lata* challenge. It indicates that all three cultivars were able to perceive the fungus, thus validating the millicell infection system using grapevine leaf discs. Interestingly, the expression of *VvSTS1*, which encodes the first enzyme of the stilbenoid pathway, was positively correlated with the level of tolerance, whereas the accumulation of the related stilbenes was not different among cultivars. Previous reports showed that an induction of *VvSTS1* gene expression and phenolic compound accumulation in grapevine cell culture treated with *E. lata* culture filtrate (Mutawila et al., 2017). Our results further suggest that stilbenes contribute to general defense to pathogens but do not have a crucial role in the tolerance of cultivars to *E lata*.

The successful use of the millicell infection system in studying the dialogue between grapevine foliar discs and *E. lata* mycelium open new perspectives. This method emphasizes the importance of fungal elicitors or effectors produced by the fungus. To our knowledge, information of molecules with such activity is scarce, whereas the secretome, proteome and toxins produced by *E. lata* and other grapevine trunk fungi have been extensively studied (Mahoney et al., 2003; Andolfi et al., 2011; Morales-Cruz et al., 2015; Masi et al., 2018). As an example, *E. lata* produces secondary metabolites, mainly acetylic and heterocyclic compounds. Toxins, such as eutypine, was determined as the main phytotoxins secreted by the fungus but the phytotoxicity of *E. lata* is probably caused by many structurally related compounds, which are responsible for leaf symptom development but have no eliciting activity (Bertsch et al., 2013). Because the millicell infection system allows the free diffusion of secreted molecules produced by both partners across a semi-permeable membrane, it opens up the possibilities to identify fungal elicitors or effectors, that are largely unknown so far, and to study temporal induced responses from either the host or the fungus. Many artificial infection methods have been developed to address questions and issues about esca disease, black dead arm and other wood decay diseases (Reis et al., 2019). The millicell system would be particularly well adapted to the identification of fungal effectors in such complex pathosystems.

Camps et al. (2010) and Mutawila et al. (2017) gave an overview of the expression patterns of genes related to defense (PR-proteins) and phenylpropanoid pathway in Cabernet Sauvignon and Dauphine cultivars, respectively, showing a differential induction of several *PR*-encoding genes, such as osmotin (*VvPR5*), β-1,3-glucanase (*VvPR2*), chitinases (*VvPR3*, *VvPR4*), and *VvPAL*. In the present study, we expanded this analysis to Merlot, Cabernet Sauvignon, and Ugni Blanc cultivars and compared the expression of different classes of *PR* and *VvPAL* genes using the millicell system. Strikingly, Merlot, which was relatively tolerant, responded to *E. lata* by inducing *VvPAL* expression and a large number of *PR* genes, belonging to different classes. In contrast, none of these defense-related genes were up-regulated upon challenge with *E. lata* in the highly susceptible cultivar Ugni Blanc. Cabernet Sauvignon exhibited intermediate responses in accordance with its moderate degree of susceptibility. Genes encoding PR proteins that were up-regulated in Merlot are described to have direct antifungal activities, such as osmotin, ribonuclease-like protein, or act through hydrolytic activities on pathogen cell wall (β-1,3-glucanase or chitinases), and represent important PR families classically induced in response to fungi (van Loon et al., 2006; Chong et al., 2008). Therefore, it may be hypothesized that the difference in susceptibly between the three cultivars could be explained, at least partly, by the differential expression of genes involved in antifungal defense and the activation of the phenylpropanoid pathway.

In *A. thaliana*, roles of SA, JA, and ET in regulating the pathogen-induced transcriptional reprograming are well known (Glazebrook, 2005). The resistance to biotrophic pathogens and the SAR is dependent on the SA pathway, which controls the expression of *PR1*, *PR2* (β-1,3-glucanase) and *PR5* (thaumatin and osmotin-like) genes. However, a functional JA- and/or ET-dependent pathway is required for the expression of the defensin *PDF1.2*, *PR3*, and *PR4* (chitinases) genes and the resistance to necrotrophic pathogens. In grapevine, while the induction of defense-related gene has been described in number of studies, the signaling pathways and the regulatory components controlling defense-related gene expression and pathogen resistance are not completely deciphered (Kortekamp, 2006; Chong et al., 2008; Dufour et al., 2013). In response to *E. lata*, Merlot, and Cabernet Sauvignon show an induction of lipoxygenase genes, *VvLOX9* and *VvLOX11* coding for enzymes of the biosynthesis of JA, and *VvPAL* gene, that encodes the first enzyme of the phenylpropanoid pathway involved in the biosynthesis production of SA. It indicates that both JA and SA signaling pathways may be activated in these cultivars. In grape, no marker genes of SA and JA/ET pathway have been clearly defined. However, parallel exists between *PR* gene expression in grape and *A. thaliana* homologs. Consequently, the up-regulation of class I chitinase (*VvPR3*), defensin (*VvPR12*), protease inhibitor (*VvPR6*), and 9-lipoxygenase (*VvLOX9*) on one side and the up-regulation of β-1,3-glucanase (*VvPR2*), osmotin and thaumatin-like (*VvPR5*), and class III chitinase (*VvPR8*) on the other side, suggests a much intense activation of JA and SA pathways in Merlot than in Cabernet Sauvignon, which probably promote the tolerance to *E. lata* (van Loon et al., 2006; Chong et al., 2008; Dufour et al., 2013; Mutawila et al., 2017). The induction of chitinase and glucanase genes in tolerant Merlot may result in the hydrolysis of fungal cell wall components and the release of β-1,3 glucans and chitin fragments. These oligosaccharides are effective elicitors, which amplify plant defense responses, protecting susceptible grapevine cultivar against several diseases, such as downy mildew (*P. viticola*) and gray mold (*B. cinerea*) (Aziz et al., 2003; Trouvelot et al., 2008). According to our results, Ugni Blanc failed to trigger such defense responses, which is in correlation with its high susceptibility. It should be interesting to monitor the temporal accumulation of PR transcripts as well as stilbenic compounds to determine whether the kinetic of these responses is important (Lambert et al., 2013). It is difficult to presume that Ugni Blanc do not produce basal levels of JA or SA. Future works including the measurement of both hormones would help to have a better comprehension of the involvement of these pathways in the tolerance/susceptibility to *E. lata*. To go further with the regulation of molecular responses of the three cultivars, we used BTH, an analog of SA, known to be an inducer of SAR and to strengthen plant defense responses to several pathogens (Friedrich et al., 1996; Dufour et al., 2013). Our data indicate that all BTH-induced genes in Merlot are not responsive to this molecule in Ugni Blanc. These results further support the hypothesis that components of the SA signaling or perception are compromised in Ugni Blanc.

Recent reports have pointed out the importance of the plant carbon partitioning in various plant pathogen-interaction systems (Naseem et al., 2017). We investigated the involvement of sugar transport and cleavage processes in grapevine cultivars challenged with *E. lata*. We monitored the expression of three types of invertase, such as *VvcwINV*, which encodes for the only annotated cell wall invertase in grape genome, and genes encoding cytosolic invertase (*VvCIN2*) and vacuolar invertase (*VvGIN2*) (Hayes et al., 2010; Barnes and Anderson, 2018). We also studied the transcript accumulation of *VvHT5*, which encodes a high affinity hexose transporter of the plasma membrane (Hayes et al., 2007, 2010). There are six expressed *VvHTs* in grape displaying various expression patterns depending on specific organs and environmental conditions (Afoufa-Bastien et al., 2010; Lecourieux et al., 2013). Accordingly, we selected VvHT5 as candidate because it was the closest homolog of AtSTP13 (82% of sequence similarity). These homologs are both inducible by several pathogens and are coordinately expressed during pathogenesis with cell wall invertases *VvcwINV* and *AtCWIN1*, respectively (Hayes et al., 2010; Lemonnier et al., 2014; Veillet et al., 2016, 2017; Yamada et al., 2016). We showed that expression of cytosolic and vacuolar invertase genes, as well as their related activities, did not show relevant modification among cultivars. Transcript levels of *VvHT5* and *VvcwINV* under *E. lata* challenge were clearly correlated with the degree of susceptibility of all three cultivars. Accordingly, recent studies have reported that the hexose transporter of the plasma membrane AtSTP13 and the cell wall invertase AtCWIN1 participate to promote resistance to extracellular pathogens by retrieving apoplastic sugars at the plant/pathogen interface (Lemonnier et al., 2014; Yamada et al., 2016). We went further and analyzed the cell wall activities in *E. Lata*-elicited leaf disc extracts. The apoplastic sucrose-degrading activity was increased in Merlot only. Considering the role of cell wall invertase in regulating the sink strength (Ruan et al., 2010; Lemoine et al., 2013; Proels and Huckelhoven, 2014; Tauzin and Giardina, 2014; Veillet et al., 2016), we hypothesized that enhanced extracellular sucrolytic machinery and hexose uptake may participate to the transition from source to sink upon infection. During infection processes, *E. lata* is restricted to the trunk and may represent an additional sink and modify source/sink relationship through the plant. Capacity of the plant to face with the reduction of source activity (decrease of photosynthesis) and the probable re-routing of photoassimilates is a crucial issue for the outcome of the grapevine/*E. lata* interaction, particularly for the transition from the asymptomatic to the symptomatic phase. By regulating the partitioning of carbon, the expression/activity of cell wall invertases and sugar transporters may play important roles in this process. In Merlot, which is a relatively tolerant cultivar, such modification of the source/sink relationship and sugar fluxes throughout the host plant may affect the availability of sugar resources for either host and the fungus and impact positively, as energy and/or signaling molecule (Berger et al., 2007; Ruan, 2014), the outcome of the interaction toward the host. These hypotheses could be explored by monitoring carbon fluxes throughout the infected plants using radiolabeled sugars. Another challenge would be to decipher the specific roles of the recently discovered sugar transporters of the SWEET (Sugar Will be Eventually Exported Transporters) family. Members of this family are described as sugar efflux facilitators. Because they are targeted by pathogen effectors, they may contribute to sugar leakage toward the apoplast and may promote pathogen growth (Chen et al., 2010; Chandran, 2015).

In the present study, we analyzed the molecular responses of three *V. vinifera* cultivars after challenge with *E. lata* using the millicell infection system, which is a rapid, efficient and highly reproducible method. This work provides new insights into the mechanisms of tolerance/susceptibility of grapevine cultivars. Our results notably highlight the importance of the intensity of the induced-responses deployed by the host and the emerging role of sugar transport and cleavage in disease tolerance. In the future, this work will be extended to a genome wide gene expression analysis including the comparison of a larger number of cultivars and several fungal strains with different levels of virulence. Altogether, this work should identify a more comprehensive set of target genes and open new perspectives to improve the tolerance of *V. vinifera* to the eutypa dieback, and more largely to wood decay diseases, which cause severe economic losses in vineyards worldwide.

## Data Availability

The raw data supporting the conclusions of this manuscript will be made available by the authors, without undue reservation, to any qualified researcher.

## Author Contributions

PC-T, GF, SLC, CC, and GM conceived and designed the experiments. CC, GM, CG, and PL carried out the experiments. SLC, PC-T, CC, GM, and CV analyzed the experiments. CC, CG, GM, CV, and PL contributed the materials and analysis tools. SLC, CC, PC-T, and CV wrote the manuscript. GF and PC-T acquired funding.

## Conflict of Interest Statement

The authors declare that the research was conducted in the absence of any commercial or financial relationships that could be construed as a potential conflict of interest.

## Footnotes

1 www.graphpad.com
