# Supplementary material for: Comparison of the Molecular Responses of Tolerant, Susceptible and Highly Susceptible Grapevine Cultivars During Interaction With the Pathogenic Fungus Eutypa lata
Source: Front Plant Sci. 2019 Jul 30;10:991. doi: 10.3389/fpls.2019.00991 (PMC6690011; doi:10.3389/fpls.2019.00991)
Supplement: Supplementary file 1 [file Data_Sheet_1.PDF]

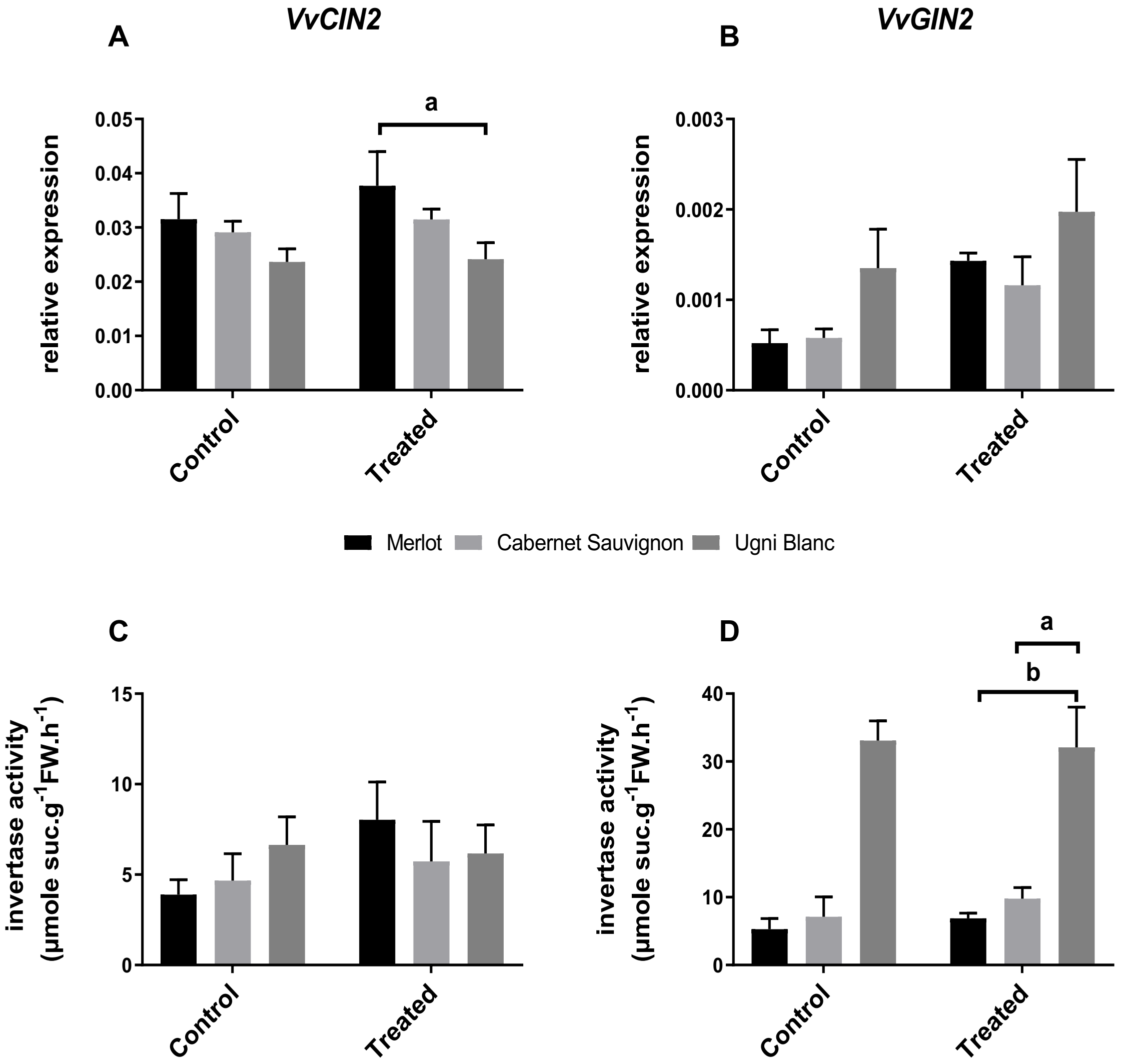

Supplementary material 1: Invertase gene expression and activities in response to E. lata infection in Merlot, Cabernet Sauvignon and Ugni Blanc cultivars. (A) Cytosolic invertase 2 (VvCIN2) and (B) vacuolar invertase 2 (VvGIN2) relative gene expression were analyzed by RT-qPCR in Merlot, Cabernet Sauvignon and Ugni Blanc leaf discs in control and treated (elicited) conditions using the Millicell system. The cytosolic (C) and vacuolar (D) invertase activities were studied in control and treated foliar discs. Data represent mean (+/- SEM) of 6 independent experiments for the invertase activity analysis and 10 independent experiments for the gene expression analysis. Statistical analysis was performed using GraphPad Prism 7.00. Stars represents significant difference between control and treated condition for each cultivar, with a Sidak's multiple comparisons test (** p-value < 0.01 and *** p-value < 0.0001). Letters represent results of a Tukey's multiple comparison test used to compare cultivars.
