# Supplementary material for: Comparison of the Molecular Responses of Tolerant, Susceptible and Highly Susceptible Grapevine Cultivars During Interaction With the Pathogenic Fungus Eutypa lata
Source: Front Plant Sci. 2019 Jul 30;10:991. doi: 10.3389/fpls.2019.00991 (PMC6690011; doi:10.3389/fpls.2019.00991)
Supplement: Supplementary file 2 [file Data_Sheet_2.PDF]

Supplementary material 2: Gene description and primer sequences used in RT-qPCR analysis.

Family		gene name	Annotation	Primer F	Primer R	Gene ID (NCBI)	References
housekeeping gene		*VvGAPDH*	glyceraldehyde-3-phosphate deshydrogenase	CCACAGACTTCATCGGTGACA	TTCTCGTTGAGGGCTATTCCA	100233024	
Sugar metabolism		*VvHT5*	hexose transporter	TAGTGATGCGTCCCTCTACTC	CTTCCAGCAAGAGCAATCGAC	100232950	
		VvcwINV	cell wall invertase 1	ACGAATCATCTAGTGTGGAGCAC	CTTAAACGATATCTCCACATCTGC	100232951	Hayes *et al*, 2010
		VvGIN2	vacuolar invertase 2	GGGGTTCTTCATGCTGTCCC	TGCTTGACTCCGGGACCATT	100241232	
		VvCIN2	neutral invertase (cytoplasmic) 2	TGGAGGACCCTTCCCATCTG	TTGGAATTTGGAAGAAGTGTGTTC	100253759	
plant defenses	phenylpropanoids	*VvPAL*	phenylalanine ammonia lyase	TGCTGACTGGTGAAAAGGTG	CGTTCCAAGCACTGAGACAA	100853659	
		VvSTS1	stilbene synthase 1	AGGGAAGCAGCATTGAAGGC	CGGGCATTTCTACACCGGAG	100217471	
	enzymes	*VvLOX9*	lipoxygenase 9	GACAAGAAGGACGAGCCTTG	CATAAGGGTACTGCCCGAAA	100241150	Dufour *et al*, 2013
		VvLOX11	lipoxygenase 11	TGCTCTACCCCACAAGCGAA	AGCAGTGTGCTCATGATTTTCCAG	100232917	
	PR proteins	*VvPR2*	β 1.3 Glucanase class I	GCAGTCGGGAACGAAGTGAG	ATGGAGGGTAGGAGTTGCCC	100232986	
		VvPR3	chitinase class I	GGGGTTGTGGGCATTGGTAG	CCCTCCACATTGCTCTGCTG	100232985	
		VvPR5	Thaumatin-like	CCTACACAGTTTGGGCAGCA	TCCCAGGGTTCACAGTCGAG	100254732	
		VvPR6	protease inhibitor-like	GCAGAAACCATTAAGAGGGAGA	TCTATCCGATGGTAGGGACACT	100264939	
		VvPR8	chitinase class III	ATCATCGTCTCGGCCATTAG	AGAGCAGTGCCCATGAACTT	100251796	
		VvPR10	ribonuclease-like	GCTCAAAGTGGTGGCTTCTC	CTCTACATCGCCCTTGGTGT	100246525	Dufour *et al*, 2013
		VvPR12	defensin-like	GACGAAAGCCACGGCAATTT	GTGTATTTTGGCAGAGATGGGC	100242603	
